# Prevalence of Short Peer Reviews in 3 Leading General Medical Journals

**DOI:** 10.1001/jamanetworkopen.2023.47607

**Published:** 2023-12-14

**Authors:** Pascal Geldsetzer, Markus Heemann, Pauli Tikka, Grace Wang, Marika Mae Cusick, Ali Lenjani, Nandita Krishnan

**Affiliations:** 1 Division of Primary Care and Population Health, Department of Medicine, Stanford University, Stanford, California; 2 Chan Zuckerberg Biohub–San Francisco, San Francisco, California; 3 Department of Development Economics, Centre for Modern Indian Studies, University of Goettingen, Göttingen, Germany; 4 Institute of Biotechnology, University of Helsinki, Helsinki, Finland; 5 Department of Biology, Stanford University, Stanford, California; 6 Department of Health Policy, Stanford University, Stanford, California

## Abstract

**Importance:**

High-quality peer reviews are often thought to be essential to ensuring the integrity of the scientific publication process, but measuring peer review quality is challenging. Although imperfect, review word count could potentially serve as a simple, objective metric of review quality.

**Objective:**

To determine the prevalence of very short peer reviews and how often they inform editorial decisions on research articles in 3 leading general medical journals.

**Design, Setting, and Participants:**

This cross-sectional study compiled a data set of peer reviews from published, full-length original research articles from 3 general medical journals (*The BMJ*, *PLOS Medicine*, and *BMC Medicine*) between 2003 and 2022. Eligible articles were those with peer review data; all peer reviews used to make the first editorial decision (ie, accept vs revise and resubmit) were included.

**Main Outcomes and Measures:**

Prevalence of very short reviews was the primary outcome, which was defined as a review of fewer than 200 words. In secondary analyses, thresholds of fewer than 100 words and fewer than 300 words were used. Results were disaggregated by journal and year. The proportion of articles for which the first editorial decision was made based on a set of peer reviews in which very short reviews constituted 100%, 50% or more, 33% or more, and 20% or more of the reviews was calculated.

**Results:**

In this sample of 11 466 reviews (including 6086 in *BMC Medicine*, 3816 in *The BMJ*, and 1564 in *PLOS Medicine*) corresponding to 4038 published articles, the median (IQR) word count per review was 425 (253-575) words, and the mean (SD) word count was 520.0 (401.0) words. The overall prevalence of very short (<200 words) peer reviews was 1958 of 11 466 reviews (17.1%). Across the 3 journals, 843 of 4038 initial editorial decisions (20.9%) were based on review sets containing 50% or more very short reviews. The prevalence of very short reviews and share of editorial decisions based on review sets containing 50% or more very short reviews was highest for *BMC Medicine* (693 of 2585 editorial decisions [26.8%]) and lowest for *The BMJ* (76 of 1040 editorial decisions [7.3%]).

**Conclusion and Relevance:**

In this study of 3 leading general medical journals, one-fifth of initial editorial decisions for published articles were likely based at least partially on reviews of such short length that they were unlikely to be of high quality. Future research could determine whether monitoring peer review length improves the quality of peer reviews and which interventions, such as incentives and norm-based interventions, may elicit more detailed reviews.

## Key Points

**Question:**

How often do very short peer reviews inform initial editorial decisions at 3 leading general medical journals?

**Findings:**

This cross-sectional study of 11 466 peer reviews corresponding to 4038 published articles found that 20.9% of initial editorial decisions were based on review sets in which at least one-half of the peer reviews had fewer than 200 words.

**Meaning:**

In this study of 3 leading general medical journals, one-fifth of initial editorial decisions for published articles were based on review sets in which very short reviews constituted at least one-half of the reviews. Under the assumption that very short reviews indicate low review quality, this finding implies that editors at these journals frequently made an initial decision based on a review set that contains a high proportion of low-quality reviews.

## Teaser

This cross-sectional study examines the prevalence of very short peer reviews (<200 words) from 3 general medical journals as a measure of peer review quality.

## Introduction

For many decades, peer review has been the key process on which the scientific community has relied to ensure that the reporting of research findings meets minimum quality standards.^1^ Peer review has become an established part of the scientific publication process and is used by most journals worldwide.^2,3^ Despite its widespread use, several deficiencies of the peer review process have been noted, such as the time burden on researchers, high costs, lengthy duration, and biases.^4,5,6,7^ Notwithstanding these deficiencies, a majority of researchers consider peer review important to ensure the quality and integrity of science.^8^

The effectiveness of peer review is contingent on its quality; however, it is unclear how prevalent low-quality reviews are and to what extent they inform editorial decisions on research articles. Likely reasons for the paucity of research in this area are the difficulty of measuring peer review quality and the lack of consensus on what constitutes quality.^8^ To date, the most commonly used approaches to evaluate peer review quality are editor and review quality evaluations.^9^ These evaluations typically consist of one or more items rated using Likert scales. Several scales have been developed but their validity has not been established.^9^

Review volume (ie, word count) could be a simple, objective metric for peer review quality. A common recommendation in guides for conducting peer review^10,11,12^ is to provide comments that are sufficiently detailed. As such, a minimum number of words is required to provide constructive feedback. Review volume is certainly not a perfect measure of peer review quality. It is possible that a manuscript is of such outstanding quality that a reviewer may not feel the need to provide more detailed comments. Conversely, a review that is long may not necessarily be a high-quality review if it lacks substantive content. Nevertheless, our study is based on the premise that whether or not a review is very short holds some valuable information in reflecting review quality.^13,14^ This premise is supported by research by Yadav,^15^ who found that review volume was positively associated with overall review quality. Additionally, a recent study^16^ found that lengthier reviews were positively associated with the number of citations received by the article (arguably a measure of article quality).

Thus, under the assumption that a very low word count reflects, at least to some degree, the quality of a peer review, this study aimed to determine the prevalence of very short peer reviews in 3 leading general medical journals:* The BMJ*, *PLOS Medicine*, and *BMC Medicine*. We defined very short reviews as having fewer than 200 words in our primary analyses, which is based on the rationale that a review that is shorter than a typical abstract is unlikely to provide constructive feedback. However, because there is no objective criterion to define such a word count threshold, we also used additional cutoffs of fewer than 100 words and fewer than 300 words in secondary analyses. We further aimed to determine the proportion of published research articles in these 3 journals for which initial editorial decisions were made on the basis of very short peer reviews. We focused on leading general medical journals, reasoning that if high-tier journals must often rely on low-quality reviews, lower-tier journals are likely to do so even more frequently.

## Methods

### Study Design and Data Sources

This cross-sectional study followed the Strengthening the Reporting of Observational Studies in Epidemiology (STROBE) reporting guideline. Because the study used publicly available data in accordance with the Common Rule, it was not considered human participants research by the Stanford University institutional review board and did not require approval or informed consent. We selected all journals in the *Medicine, General and Internal* category of the Clarivate journal citation reports that were among the 20 journals with the highest 2020 journal impact factor and had published peer review reports of at least a subset of full-length research articles that appeared in the journal.^17^ We used the 2020 journal impact factor to avoid the possibly transient effects of the COVID-19 pandemic on journal rankings by impact factor. Three journals met these criteria: *The BMJ*, *BMC Medicine*, and *PLOS Medicine*. *The BMJ *has a fully open peer review process; it publishes the peer review history and reviewer names for all published research articles*. BMC Medicine* runs a peer review process in which the peer review history is published but reviewers are given the choice to disclose their identity. *PLOS Medicine* lets authors opt in to publish the peer review history of their accepted manuscript and discloses reviewer names only if reviewers choose to sign their review. All 3 journals publish the prepublication history of accepted manuscripts, which includes all previous versions of the manuscript, reviewer comments, and authors’ responses.^18,19,20^ Only *The BMJ* provides the self-reported job title of the reviewer.

Articles included in this study were published between January 2003 and December 2022. *The BMJ* began publishing all peer reviews of published articles in 2015. *BMC Medicine* began publishing peer review history in 2003. *PLOS Medicine* began allowing authors to opt in in 2019. The most recent impact factors (2021) of these journals were: 96.21 (*The BMJ*), 11.81 (*BMC Medicine*), and 11.61 (*PLOS Medicine*).^21^ We restricted our analysis to peer reviews of published, full-length, original research articles because word count cutoffs for defining quality may be different for peer reviews of shorter research works, such as brief reports and rejected articles. Only the first set of peer reviews that an article received (ie, reviews used to arrive at an initial editorial decision of accept or revise and resubmit) was included in the analysis. We included all articles for which peer review data were available. If peer reviews for an article were made available, all peer reviews that the article received (rather than a subset) were made available. For *PLOS Medicine* and for *BMC Medicine* all peer reviews were available for a subset of all published articles. No further subsampling was applied for *PLOS Medicine*. For *BMC Medicine*, we compared results in 2-year aggregate levels and found similar patterns for early years in which only a subset of articles had published peer review history and subsequent years in which all articles had published peer review histories. We developed code in Python version 3.7 (Python Software Foundation) to count the words of each published peer review. The scraping from the journal websites was performed between November 2022 and January 2023.

### Definition of Very Short Peer Reviews

In our primary analysis, we defined very short peer reviews as those containing fewer than 200 words. Although there is no objective criterion to inform the selection of a word count threshold that best reflects peer review quality, we chose the cutoff of fewer than 200 words for the following reasons. Given that the 3 journals have abstract lengths between 250 words and 350 words, we believe that reviews that are substantially shorter than a high-level manuscript summary are unlikely to provide sufficiently detailed feedback. Additionally, established instruments for measuring the quality of peer review, such as the *The Journal of Bone and Joint Surgery* Peer Review Scoring Scale,^22^ have used 200 words as the minimum word count threshold that corresponds to the highest scoring category. Nevertheless, we recognize that there is some subjectivity to selecting this cutoff and therefore, also present results using cutoffs of fewer than 100 words and fewer than 300 words.

### Statistical Analysis

We created plots to examine the distribution of the number of words across peer reviews and the number of reviews across research articles for all journals and years combined. We also calculated the distribution (mean [SD] and median [IQR]) of the number of reviews per article and words per review disaggregated by journal and year. We used the Kruskal-Wallis test to assess significant differences in median word counts across journals. To test for significant differences in mean review word counts across journals, we used 1-way analysis of variance with the Holm method to adjust for multiple comparisons.^23^ Following this, we categorized word counts of peer reviews (0-99 words; 100-199 words; 200-299 words; 300-399 words; 400-499 words; and ≥500 words) and calculated the prevalence of reviews for each category by year for each journal.

Given the importance of the first editorial decision, we analyzed the proportion of articles for which this initial decision was based on very short peer reviews (ie, reviews of <200 words). For each article, we categorized its associated peer reviews into 1 of 4 groups (that were not mutually exclusive) based on the proportion of very short reviews in the set (100% very short reviews; ≥50% very short reviews; ≥33% very short reviews; and ≥20% very short reviews).

Finally, we also plotted the distribution of peer review word count by reviewer seniority for reviews from *The BMJ*. We defined senior scientists as those whose self-reported job title contained any of the following terms: *professor* (excluding those with the addition of *assistant* or *associate*), *dean*, *head*, *chief*, *director,* or *chair*. All other reviewers were classified as junior scientists. A 2-sided *P* < .05 was considered statistically significant. Statistical analyses were conducted using Stata statistical software version 16.0 (StataCorp).

## Results

**Figure 1.**
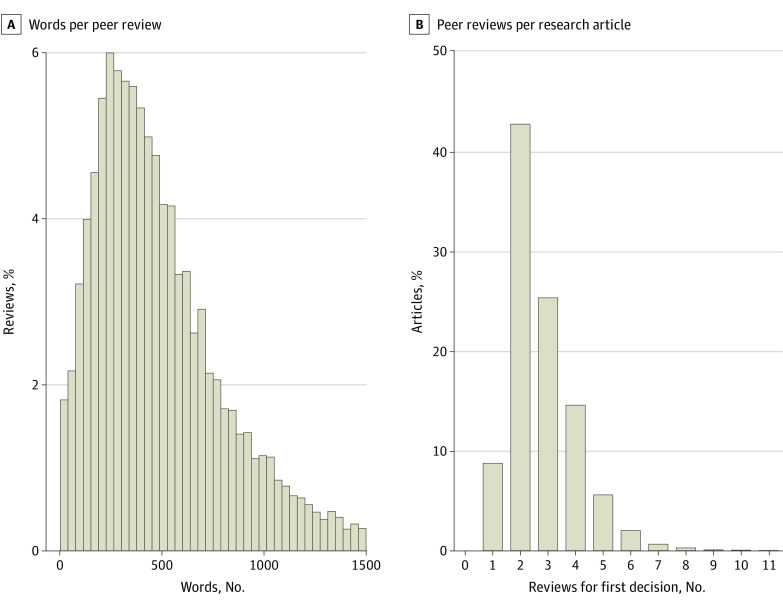
Distribution of the Number of Words per Peer Review and the Number of Peer Reviews per Research Article for All Years and Journals Combined The chart shows the distribution of the number of words per peer review (A) and the number of reviews per article (B). Reviews with a word count of more than 1500 words (325 of 11 466 reviews [2.8%]) are not shown. No articles received more than 9 peer reviews.

Our search yielded a total of 11 466 peer reviews corresponding to 4038 published articles, including 6086 reviews in *BMC Medicine*, 3816 in *The BMJ*, and 1564 in *PLOS Medicine*. The distribution of the number of peer reviews per article showed that 1725 of 4038 articles (42.7%; 95% CI, 41.2%-44.2%) received 2 reviews (Figure 1). A notable exception was *PLOS Medicine*, where 206 of 413 articles (49.9%; 95% CI, 45.0%-54.7%) received 4 initial reviews (eFigure 1 in Supplement 1). Regarding the distribution of the number of words per review, the highest proportion of reviews had approximately 250 words (Figure 1). The distribution was right skewed, with 677 reviews (5.9%) containing fewer than 100 words; 1958 reviews (17.1%) containing fewer than 200 words; 8345 reviews (72.7%) containing between 200 words and 1000 words; and 1163 reviews (10.2%) containing more than 1000 words. Distributions of word counts per peer review and reviews per research article by journal and year are shown in eFigure 1, eFigure 2, eFigure 3, eFigure 4, and eFigure 5 in Supplement 1.

The distribution of the number of reviews per article and word count per review disaggregated by journal and year are shown in the Table. Across all 3 journals and all years analyzed, the median (IQR) word count per review was 425 (253-675) words, and the mean (SD) was 520.0 (401.0) words. *The BMJ* had the highest amount of words per review, with a median (IQR) of 525 (318-820) words and mean (SD) of 636.0 (464.4) words. *BMC Medicine* had the lowest amount of words per review, with a median (IQR) of 376 (221-590) words and mean (SD) of 455.2 (350.2) words. The Kruskal-Wallis test showed significant differences in the median review word count across all 3 journals. The *F* test for any difference in mean peer review word count across the 3 journals was also significant. In pairwise testing, differences in the mean word count between all pairs of journals were statistically significant.

**Table.** Distribution of the Number of Peer Reviews per Research Article and Word Count Per Peer Review by Journal and Year^a^

Year and measure	Median (IQR)			
	All journals	*BMC Medicine*	*The BMJ* ^b^	*PLOS Medicine* ^b^
All				
Reviews, No.	11 466	6086	3816	1564
Reviews per article^c^	3 (2-3)	2 (2-3)	4 (3-5)	4 (3-4)
Words per review	425 (253-675)	376 (221-590)	525 (318-820)	411 (254-653)
2003				
Reviews, No.	4	4	NA	NA
Reviews per article^c^	2 (2-2)	2 (2-2)	NA	NA
Words per review	416 (207-1048)	416 (207-1048)	NA	NA
2004				
Reviews, No.	65	65	NA	NA
Reviews per article^c^	2 (2-2)	2 (2-2)	NA	NA
Words per review	393 (199-580)	393 (199-580)	NA	NA
2005				
Reviews, No.	28	28	NA	NA
Reviews per article^c^	2 (1-3)	2 (1-3)	NA	NA
Words per review	270 (157-421)	270 (157-421)	NA	NA
2006				
Reviews, No.	60	60	NA	NA
Reviews per article^c^	2 (2-3)	2 (2-3)	NA	NA
Words per review	357 (256-575)	357 (256-575)	NA	NA
2007				
Reviews, No.	71	71	NA	NA
Reviews per article^c^	2 (2-3)	2 (2-3)	NA	NA
Words per review	438 (256-645)	438 (256-645)	NA	NA
2008				
Reviews, No.	37	37	NA	NA
Reviews per article^c^	2 (2-3)	2 (2-3)	NA	NA
Words per review	325 (240-580)	325 (240-580)	NA	NA
2009				
Reviews, No.	33	33	NA	NA
Reviews per article^c^	2 (2-2)	2 (2-2)	NA	NA
Words per review	380 (232-520)	380 (232-520)	NA	NA
2010				
Reviews, No.	70	70	NA	NA
Reviews per article^c^	2 (2-3)	2 (2-3)	NA	NA
Words per review	290 (165-559)	290 (165-559)	NA	NA
2011				
Reviews, No.	148	148	NA	NA
Reviews per article^c^	2 (2-3)	2 (2-3)	NA	NA
Words per review	316 (180.5-501.5)	316 (181-502)	NA	NA
2012				
Reviews, No.	221	221	NA	NA
Reviews per article^c^	2 (2-3)	2 (2-3)	NA	NA
Words per review	282 (145-470)	282 (145-470)	NA	NA
2013				
Reviews, No.	333	333	NA	NA
Reviews per article^c^	2 (2-3)	2 (2-3)	NA	NA
Words per review	313 (172-529)	313 (172-529)	NA	NA
2014				
Reviews, No.	313	313	NA	NA
Reviews per article^c^	2 (2-2)	2 (2-2)	NA	NA
Words per review	335 (198-563)	335 (198-563)	NA	NA
2015				
Reviews, No.	671	380	291	NA
Reviews per article^c^	2 (2-3)	2 (2-2)	3 (2-4)	NA
Words per review	406 (240-683)	346 (196-583)	482 (309-762)	NA
2016				
Reviews, No.	679	204	475	NA
Reviews per article^c^	2 (1-3)	1 (1-2)	3 (2-4)	NA
Words per review	538 (336-819)	404 (212-629)	576 (389-978)	NA
2017				
Reviews, No.	876	443	433	NA
Reviews per article^c^	3 (2-3)	2 (2-3)	3 (3-4)	NA
Words per review	452.5 (253.5-725.5)	368 (212-591)	566 (314-862)	NA
2018				
Reviews, No.	979	505	474	NA
Reviews per article^c^	3 (2-3)	2 (2-3)	3 (3-4)	NA
Words per review	436 (262-694)	391 (230-617)	488 (317-783)	NA
2019				
Reviews, No.	1052	486	463	103
Reviews per article^c^	3 (2-3)	2 (2-3)	3 (2-4)	4 (4-4)
Words per review	444.5 (270.5-677.5)	411.5 (260-605)	509 (298-773)	381 (241-621)
2020				
Reviews, No.	1824	828	642	354
Reviews per article^c^	3 (2-4)	2 (2-3)	4 (3-5)	4 (1-4)
Words per review	410 (239-683)	371 (218-618)	531.5 (299-794)	358 (191-557)
2021				
Reviews, No.	1941	751	527	663
Reviews per article^c^	3 (2-4)	3 (2-3)	4 (3-5)	4 (4-4)
Words per review	432 (273-670)	402 (249-579)	508 (308-798)	417 (274-672)
2022				
Reviews, No.	2061	1106	511	444
Reviews per article^c^	3 (2-4)	2 (2-3)	4 (3-5)	4 (4-5)
Words per review	440 (277-678)	411 (261-611)	517 (317-865)	452.5 (281-689)

Abbreviation: NA, not available.

^a^ Only the first set of peer reviews that an article received (ie, the peer reviews used to arrive at a first editorial decision of accept or revise and resubmit) was included in the analysis. The analysis was restricted to research articles only.

^b^ NA indicates that no open peer reviews were available for the given journal in that year.

^c^ The 3 journals did not make peer reviews openly available for all research articles published; however, if peer reviews for an article were made openly available, all peer reviews that the article received (rather than a subset) were made available.

**Figure 2.**
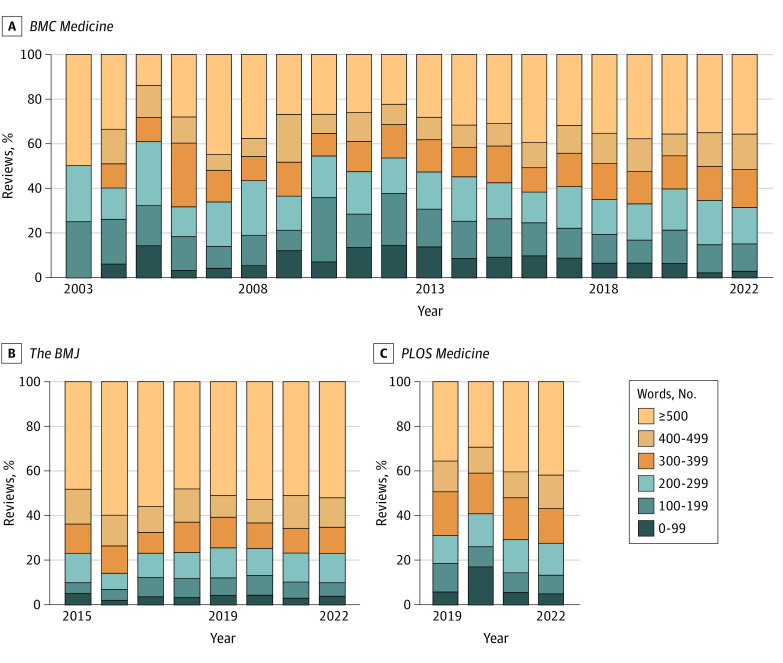
The Proportion of Peer Reviews in Different Word Count Categories by Journal and Year The figure shows the word count distributions of peer reviews for *BMC Medicine* (A), *The BMJ* (B), and *PLOS Medicine* (C).

For all journals, peer review word counts remained relatively consistent across time (Figure 2). Of the 3 journals, *BMC Medicine* had the greatest proportion of overall reviews with fewer than 200 words (1276 of 6086 reviews [21.0%]; 95% CI, 19.9%-22.0%) (eFigure 1 in Supplement 1). *The BMJ* had the lowest proportion of reviews with fewer than 200 words (417 of 3816 reviews [10.9%]; 95% CI, 9.9%-11.9%). Comparing the share of very short reviews per article by applying equal article weights rather than equal review weights yielded similar results. Across all journals, 18.6% of reviews per article (95% CI, 17.7%-19.5%) contained fewer than 200 words. This proportion was highest among *PLOS Medicine* articles, where 23.9% of reviews per article (95% CI, 20.8%-27.0%) were fewer than 200 words. For *BMC Medicine* 20.9% of reviews per article (95% CI, 19.8%-22.1%) contained fewer than 200 words and for *The BMJ*, 10.7% of reviews per article (95% CI, 9.6%-11.8%) contained fewer than 200 words.

**Figure 3.**
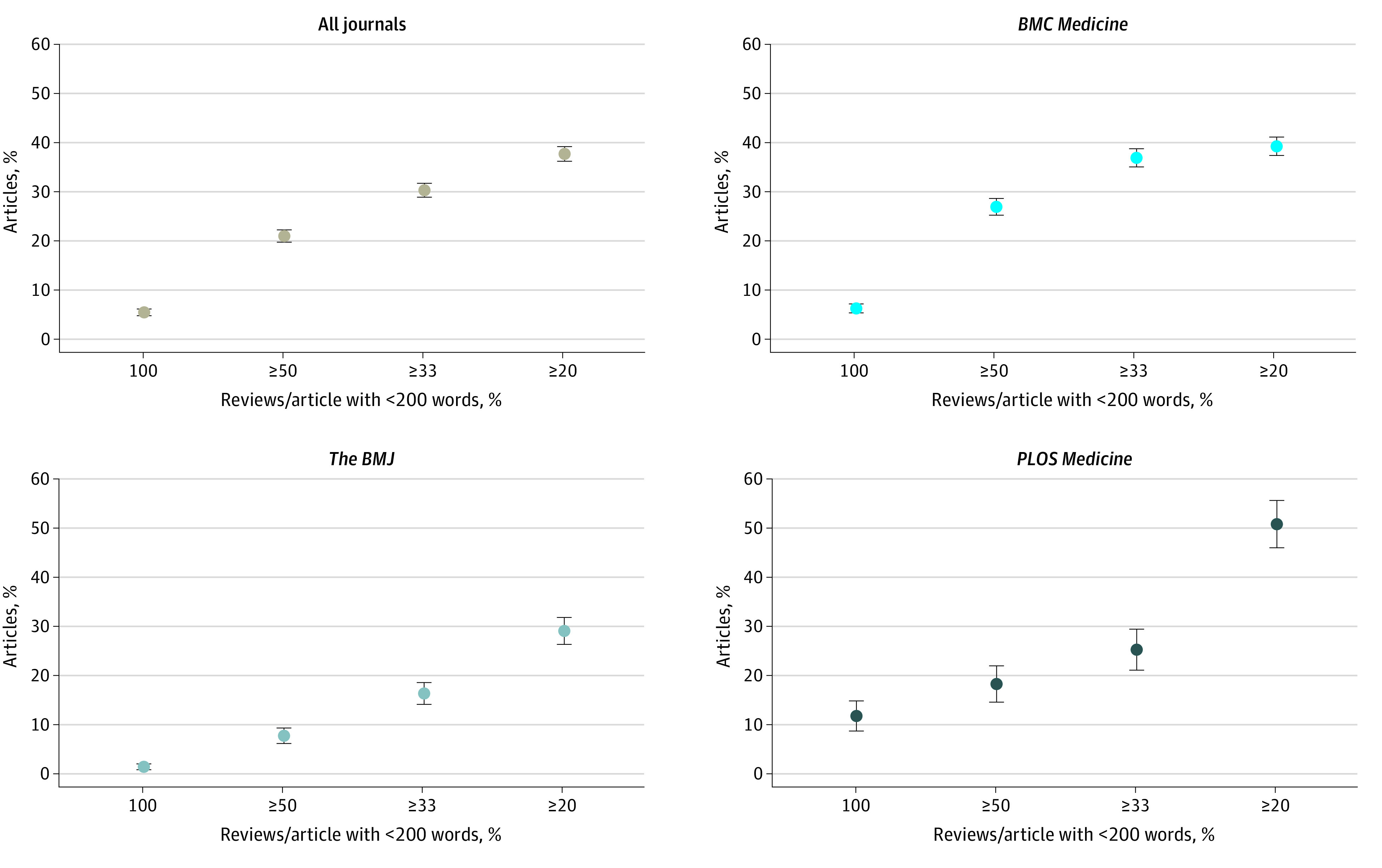
Proportion of Articles for Which the First Editorial Decision Was Based on Peer Review Sets Consisting of 100%, 50% or More, 33% or More, and 20% or More Very Short Reviews The figure shows distributions of articles (≥100%, ≥50%, ≥33%, and ≥20%) for which the first editorial decision was based on very short peer reviews for all journals, *BMC Medicine*, *The BMJ*, and *PLOS Medicine*. A very short review was defined as fewer than 200 words. The denominator for the proportion of articles is the total number of research articles that were published and for which the journal made all peer reviews openly available. Dots denote means and error bars denote 95% CIs.

Figure 3 shows the proportion of articles for which the first editorial decision was made on the basis of a set of peer reviews that consisted of 100% very short reviews, 50% or more very short reviews, 33% or more very short reviews, or 20% or more very short reviews. Across all journals, 843 of 4038 first editorial decisions (20.9%) were based on review sets containing 50% or more very short reviews. *BMC Medicine* had the highest proportion of initial editorial decisions that were based on peer review sets in which 50% or more of the reviews were very short (693 of 2585 editorial decisions [26.8%; 95% CI, 25.1%-28.5%]). *The BMJ* had the lowest proportion of editorial decisions based on peer review sets with 50% or more very short reviews (76 of 1040 editorial decisions [7.3%; 95% CI, 5.7%-8.9%]). Similar results were observed when we used cutoffs of fewer than 100 words (eFigure 6 in Supplement 1) and fewer than 300 words (eFigure 7 in Supplement 1) to define very short reviews. For reviews of fewer than 100 words, approximately 1 in 20 articles were published on the basis of an initial editorial decision that had to rely on a peer review set in which such excessively short reviews constituted at least 50% of the reviews.

**Figure 4.**
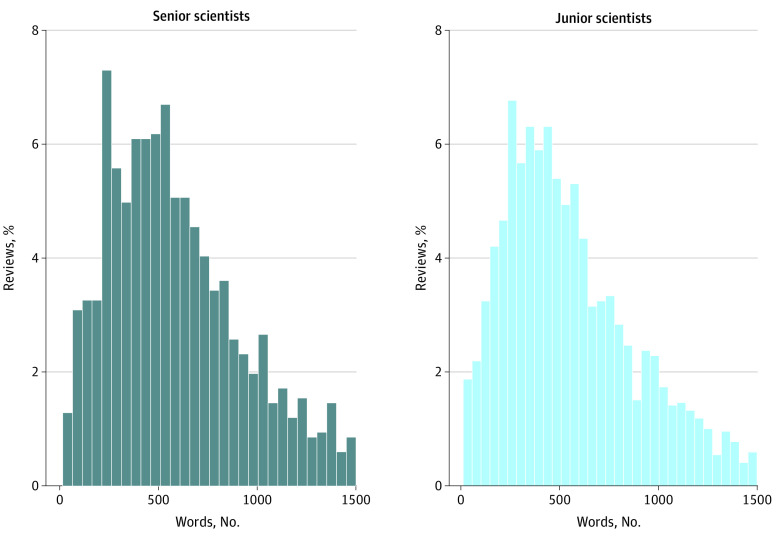
Distribution of the Number of Words per Peer Review in *The BMJ* by Reviewer Seniority This analysis of senior scientists and junior scientists was restricted to *The BMJ* (3817 peer reviews) because this was the only journal that made the self-reported job title of the peer reviewer available. For 292 reviews (7.6%), the job titles of the reviewers were missing. Senior scientists (1218 senior scientists) were defined as those self-reporting a job title containing any of the following terms: *professor* (without the addition of the words *assistant* or *associate*), *dean*, *head*, *chief*, *director*, or *chair*. Reviewers with all other job titles were considered junior scientists (2305 junior scientists). Reviews with more than 1500 words (181 reviews) were excluded in this figure.

Finally, we analyzed the distribution of the number of words per peer review by reviewer seniority for *The BMJ*. The mean (SD) number of words per review for junior scientists (631.5 [481.3] words; 95% CI, 611.8-651.2 words) and senior scientists (643.2 [443.4] words; 95% CI, 618.3-668.1 words) were similar; the proportion of reviews in different word count categories was also similar between junior and senior scientists (Figure 4).

## Discussion

In this cross-sectional study of peer reviews from 3 leading general medical journals, we found that across all journals and years, 17.1% of reviews were very short (ie, <200 words). Also, 20.9% of initial editorial decisions that led to publication were based on review sets in which very short reviews accounted for 50% or more of the reviews. Even when using a more conservative threshold (<100 words) to define very short reviews, approximately 1 in 20 articles were published on the basis of an initial editorial decision that had to rely on a peer review set in which such excessively short reviews constituted at least 50% of the reviews.

Compared with *PLOS Medicine* and *BMC Medicine*, *The BMJ* had significantly longer reviews on average and a lower share of very short reviews. Additionally, *The BMJ* had the lowest proportion of editorial decisions made based on peer review sets with 50% or more very short reviews. These results were consistent across different word count thresholds used to define very short reviews. Review word count has been shown to be positively associated with journal impact factor^8,24,25^; thus, these findings may be associated with differences in journal impact factors (journal impact factor: 96.21 for *The BMJ* vs 11.61 for *PLOS Medicine* vs 11.81 for *BMC Medicine*).^21^ However, the mechanisms underlying this association are not fully clear. It is possible that reviewers invest more effort when conducting reviews for higher impact journals.^8,25^ Similarly, it is possible that higher impact journals are able to identify willing reviewers that, on average, provide more thorough reviews.^8^

We did not find significant differences in review word count by reviewer seniority for *The BMJ*. This finding is consistent with previous research,^26^ which found that reviewer seniority was not associated with review quality. However, other reviewer characteristics for which we did not have data may also be relevant. For instance, some data suggest that reviewers from predominantly English-speaking countries tend to write longer reviews, and it is possible that higher impact journals are more likely to solicit reviewers from these countries.^8,25^

The differences in prevalence of very short peer reviews across journals in our study could also stem from differences in the design of the peer review process itself. Of the 3 journals, only *The BMJ* has a completely open peer review process, in which the reviews and names of reviewers are published by default. Additionally, based on information provided by each of the journals on their websites, it appears that *The BMJ* provides the most incentives (all nonmonetary) for reviewers (eg, article processing charge discounts, continuing medical education credits, and certificates).^27,28,29^ It is, thus, possible that increasing accountability and providing incentives are approaches that could encourage reviewers to provide more detailed reviews and that similar measures could also increase review length at other journals such as *BMC Medicine* and *PLOS Medicine*.^30,31^ Norm-based interventions have a strong evidence base for changing behaviors and could also potentially be used in this context.^32^ Future studies could investigate the impact of interventions targeting norms on peer review word count, such as posting sample reviews and indicating to reviewers how their review length compares with other reviews for similar articles.^33^

### Limitations

This study has some limitations. We reiterate that using word count as a measure of review quality, although simple to measure, has limitations that more complex techniques assessing the content of reviews for quality may not have. A review could be very short because the article had no flaws that merited critiquing. Similarly, a longer review may not necessarily be of higher quality if it does not provide competent feedback. Thus, other attributes of a review, such as its relevance and coverage, should ideally also be considered when assessing review quality.^33^ Nevertheless, word count appears to be an important component of quality and is easy to measure.^15^ We also acknowledge that our selection of a cutoff of fewer than 200 words to define very short reviews is arbitrary. Recognizing this limitation, we show all results using alternative cutoffs.

An additional limitation is that we only had access to the review history for those articles that were eventually published. Furthermore, in the case of *PLOS Medicine*, authors had to actively opt in to make the review history for their article publicly available. Thus, it is unclear whether our findings generalize to reviews of articles that were rejected. In the case of *PLOS Medicine*, authors may additionally have only opted to publish the peer review history if the reviewers had little to criticize. We also only analyzed data from 3 medical journals, which is a small nonrandom sample. However, given that our journals are high-impact general medical journals, we believe that our estimates are conservative for the medical field overall on the basis of the assumption that it is likely that lower-tier journals have a higher prevalence of very short reports and rely more frequently on these types of reports to make editorial decisions.

Furthermore, we acknowledge that using job titles as proxy for reviewer seniority may not be representative for tenure, experience, or expertise. Nonetheless, at aggregate levels, we would expect a certain degree of experience and expertise advantage, particularly in the medical space.

## Conclusions

In this cross-sectional study of prevalence of very short reviews in 3 leading general medical journals, we found that 20.9% of initial editorial decisions were made according to review sets in which reviews of fewer than 200 words constituted 50% or more of the reviews. Future studies could leverage increasingly public peer review data to investigate whether monitoring peer review length is a useful approach or what other more complex measures may improve journals’ ability to monitor and improve their peer review quality. Future research could also assess the effectiveness of incentives and norm-based interventions in eliciting more detailed reviews.

## Notes

Supplement 1. **eFigure 1.** Distribution of the Number of Words Per Peer Review and the Number of Peer Reviews Per Research Article by Journal **eFigure 2.** Distribution of the Number of Words Per Peer Review and the Number of Peer Reviews Per Research Article for *BMC Medicine* Between 2003-2008 **eFigure 3.** Distribution of the Number of Words Per Peer Review and the Number of Peer Reviews Per Research Article for *BMC Medicine* Between 2009-2014 **eFigure 4.** Distribution of the Number of Words Per Peer Review and the Number of Peer Reviews Per Research Article For *BMC Medicine* and *The BMJ* Between 2015-2018 **eFigure 5.** Distribution of the Number of Words Per Peer Review and the Number of Peer Reviews Per Research Article for *BMC Medicine*, *The BMJ*, and *PLOS Medicine* Between 2019-2022 **eFigure 6.** Proportion of Articles for Which the First Editorial Decision Was Made Based on a Set of Peer Reviews Consisting of 100%, ≥50%, ≥33%, or ≥20% of Reviews With <100 Words **eFigure 7.** Proportion of Articles for Which the First Editorial Decision Was Made Based on a Set of Peer Reviews Consisting of 100%, ≥50%, ≥33%, or ≥20% of Reviews With <300 Words

Supplement 2.
Data Sharing Statement
