# Supplementary material for: Prevalence of Short Peer Reviews in 3 Leading General Medical Journals
Source: JAMA Netw Open. 2023 Dec 14;6(12):e2347607. doi: 10.1001/jamanetworkopen.2023.47607 (PMC10722334; doi:10.1001/jamanetworkopen.2023.47607)
Supplement: Supplement 2. — Data Sharing Statement [file jamanetwopen-e2347607-s002.pdf]

Data Sharing Statement

Geldsetzer. Prevalence of Short Peer Reviews in 3 Leading General Medical Journals. *JAMA Netw Open*. Published December 14, 2023. doi:10.1001/jamanetworkopen.2023.47607

Data
Data available: Yes
Data types: Data (not involving human participants)
How to access data: https://github.com/paulitikka/three-data-extraction-examples
https://github.com/paulitikka/combining-csvs-in-many-folders-and-names-with-panda/blob/master/Combining%20Matrices_Tikka051120.py
https://github.com/paulitikka/histograms/blob/master/histograms_tikka101120.py
When available: With publication

Supporting Documents
Document types: Statistical/analytic code
How to access documents: https://github.com/paulitikka/three-data-extraction-examples
https://github.com/paulitikka/combining-csvs-in-many-folders-and-names-with-panda/blob/master/Combining%20Matrices_Tikka051120.py
https://github.com/paulitikka/histograms/blob/master/histograms_tikka101120.py
When available: With publication

Additional Information
Who can access the data: Anyone requesting the data
Types of analyses: All
Mechanisms of data availability: Github
