# Supplementary material for: Prevalence of Short Peer Reviews in 3 Leading General Medical Journals
Source: JAMA Netw Open. 2023 Dec 14;6(12):e2347607. doi: 10.1001/jamanetworkopen.2023.47607 (PMC10722334; doi:10.1001/jamanetworkopen.2023.47607)

Supplemental Online Content

Geldsetzer P, Heemann M, Tikka P, et al. Prevalence of short peer reviews in 3 leading general medical journals *JAMA Netw Open*. 2023;6(12):e2347607. doi:10.1001/jamanetworkopen.2023.47607

eFigure 1. Distribution of the Number of Words Per Peer Review and the Number of Peer Reviews Per Research Article by Journal

eFigure 2. Distribution of the Number of Words Per Peer Review and the Number of Peer Reviews Per Research Article for *BMC Medicine* Between 2003-2008

eFigure 3. Distribution of the Number of Words Per Peer Review and the Number of Peer Reviews Per Research Article for *BMC Medicine* Between 2009-2014

eFigure 4. Distribution of the Number of Words Per Peer Review and the Number of Peer Reviews Per Research Article For *BMC Medicine* and *The BMJ* Between 2015-2018

eFigure 5. Distribution of the Number of Words Per Peer Review and the Number of Peer Reviews Per Research Article for *BMC Medicine*, *The BMJ*, and *PLOS Medicine* Between 2019-2022

eFigure 6. Proportion of Articles for Which the First Editorial Decision Was Made Based on a Set of Peer Reviews Consisting of 100%, ≥50%, ≥33%, or ≥20% of Reviews With <100 Words

eFigure 7. Proportion of Articles for Which the First Editorial Decision Was Made Based on a Set of Peer Reviews Consisting of 100%, ≥50%, ≥33%, or ≥20% of Reviews With <300 Words

This supplemental material has been provided by the authors to give readers additional information about their work.

eFigure 1: Distribution of the number of words per peer review and the number of peer reviews per research article by journal

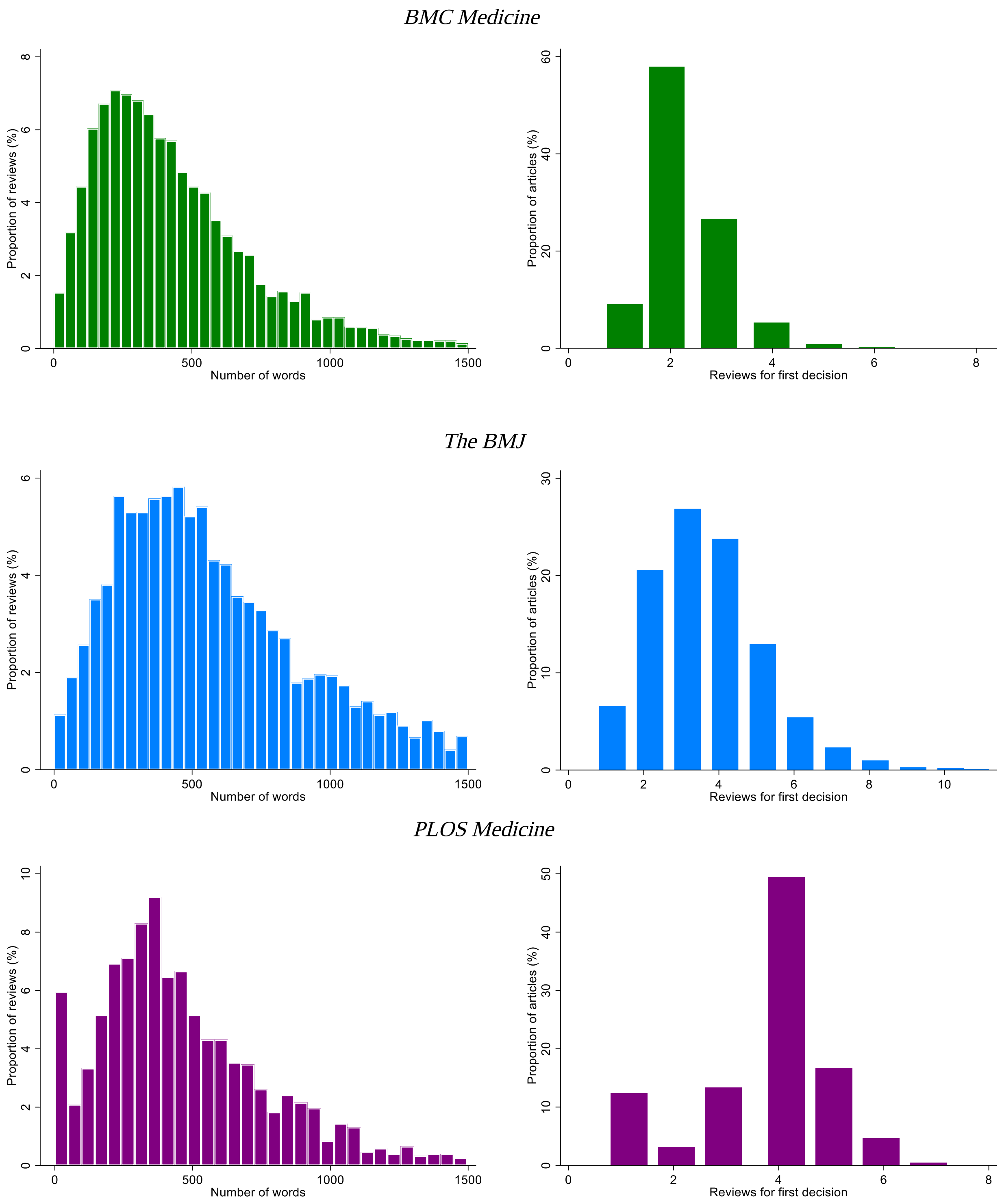

The distribution of word counts per peer review (left column of panels) and reviews per research article (right column of panels) are shown for *BMC Medicine* (green), *The BMJ* (blue), and *PLOS Medicine* (purple) from 2003-2020. Reviews with over 1500 words were excluded.

eFigure 2: Distribution of the number of words per peer review and the number of peer reviews per research article for *BMC Medicine* between 2003-2018

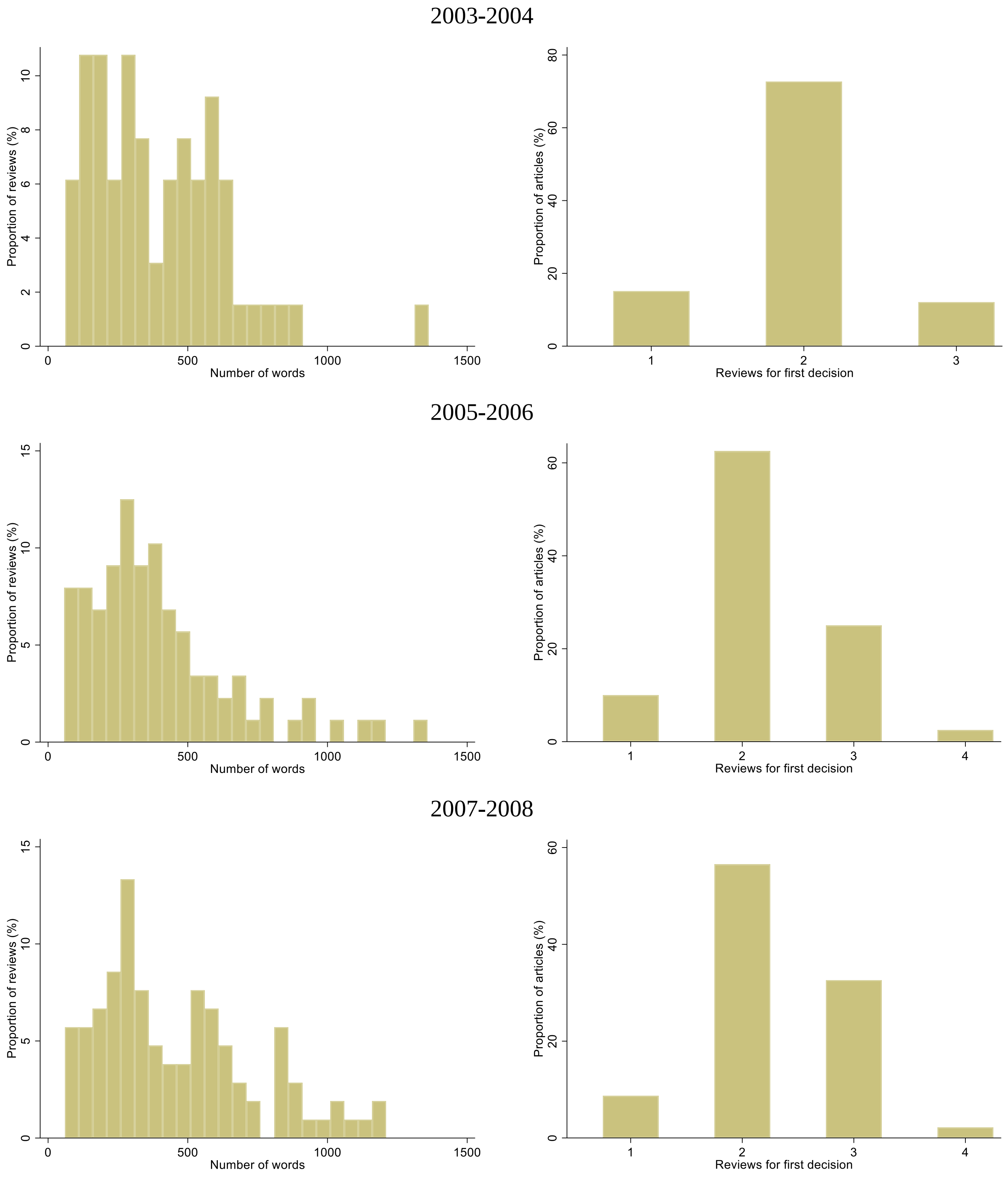

Reviews with over 1500 words were excluded.

eFigure 3: Distribution of the number of words per peer review and the number of peer reviews per research article for *BMC Medicine* between 2009-2014

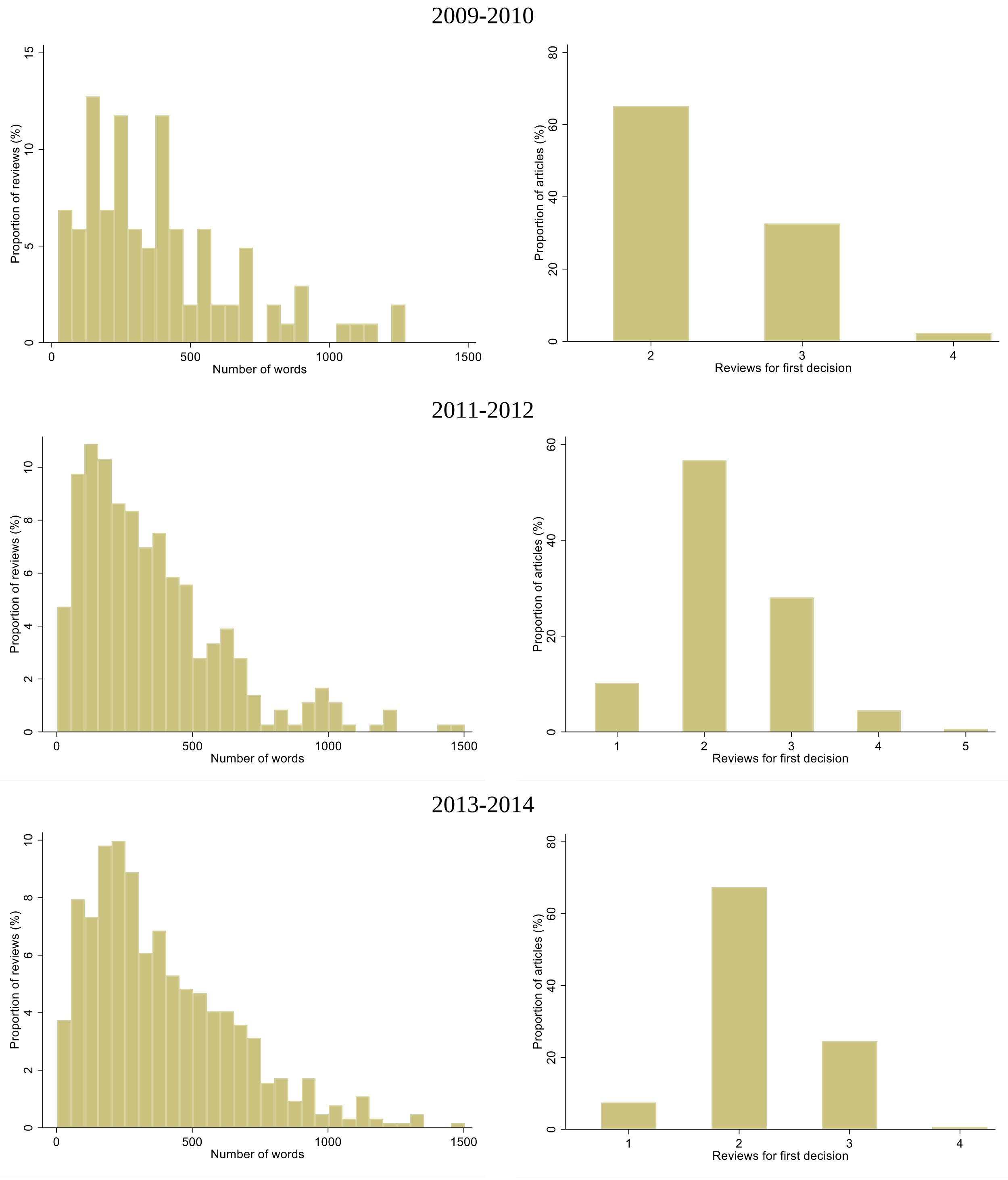

Reviews with over 1500 words were excluded.

eFigure 4: Distribution of the number of words per peer review and the number of peer reviews per research article for *BMC Medicine* and *The BMJ* between 2015-2018

2015-2016

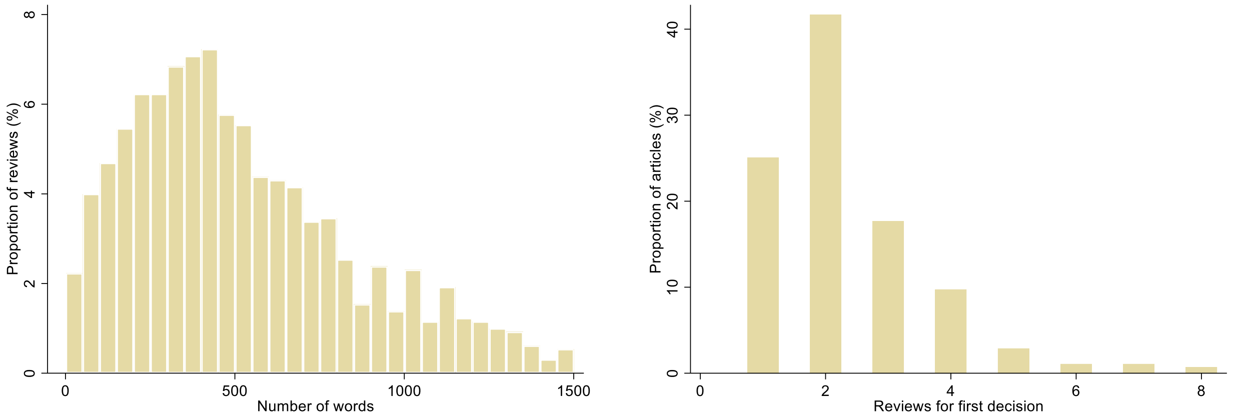

2017-2018

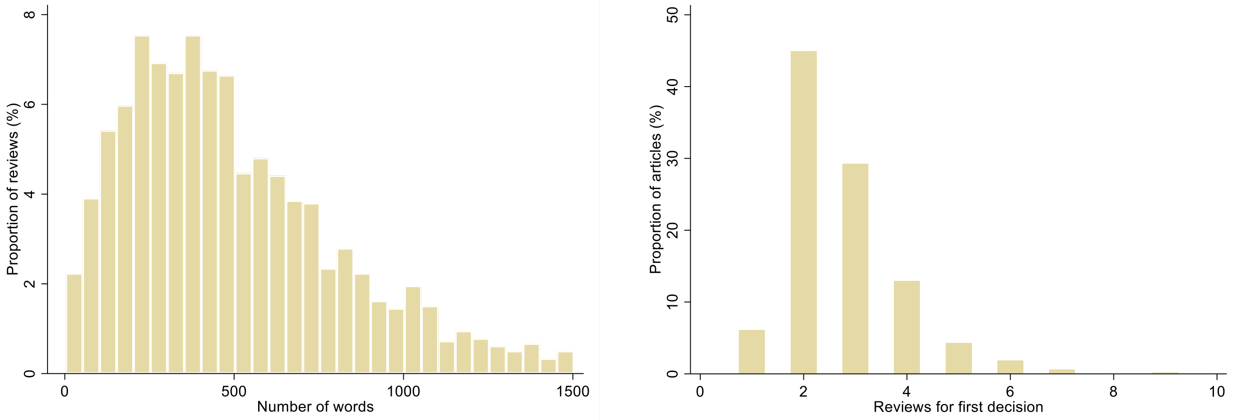

Reviews with over 1500 words were excluded.

eFigure 5: Distribution of the number of words per peer review and the number of peer reviews per research article for *BMC Medicine, The BMJ,* and *PLOS Medicine* between 2019-2022

2019-2020

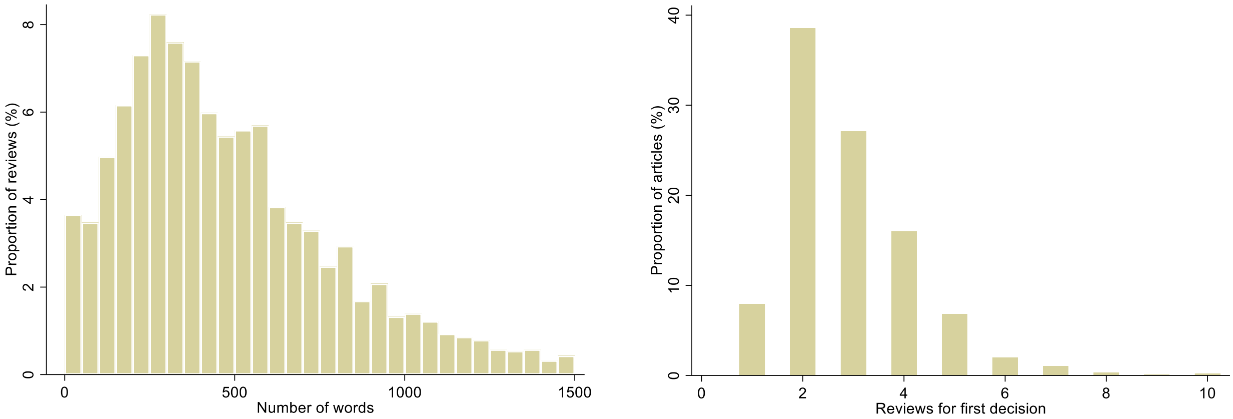

2021-2022

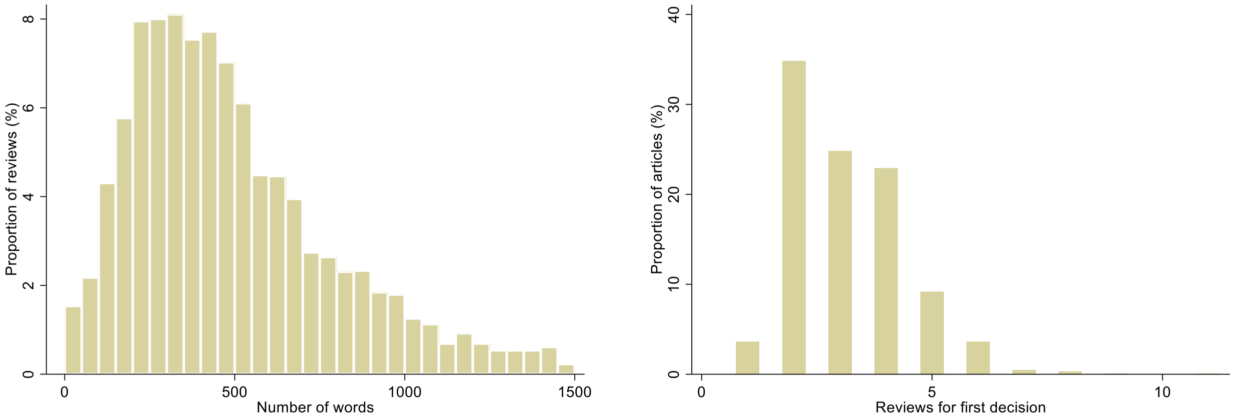

Reviews with over 1500 words were excluded.

eFigure 6: Proportion of articles for which the first editorial decision was made based on a set of peer reviews consisting of 100%, ≥50%, ≥33%, or ≥20% of reviews with <100 words

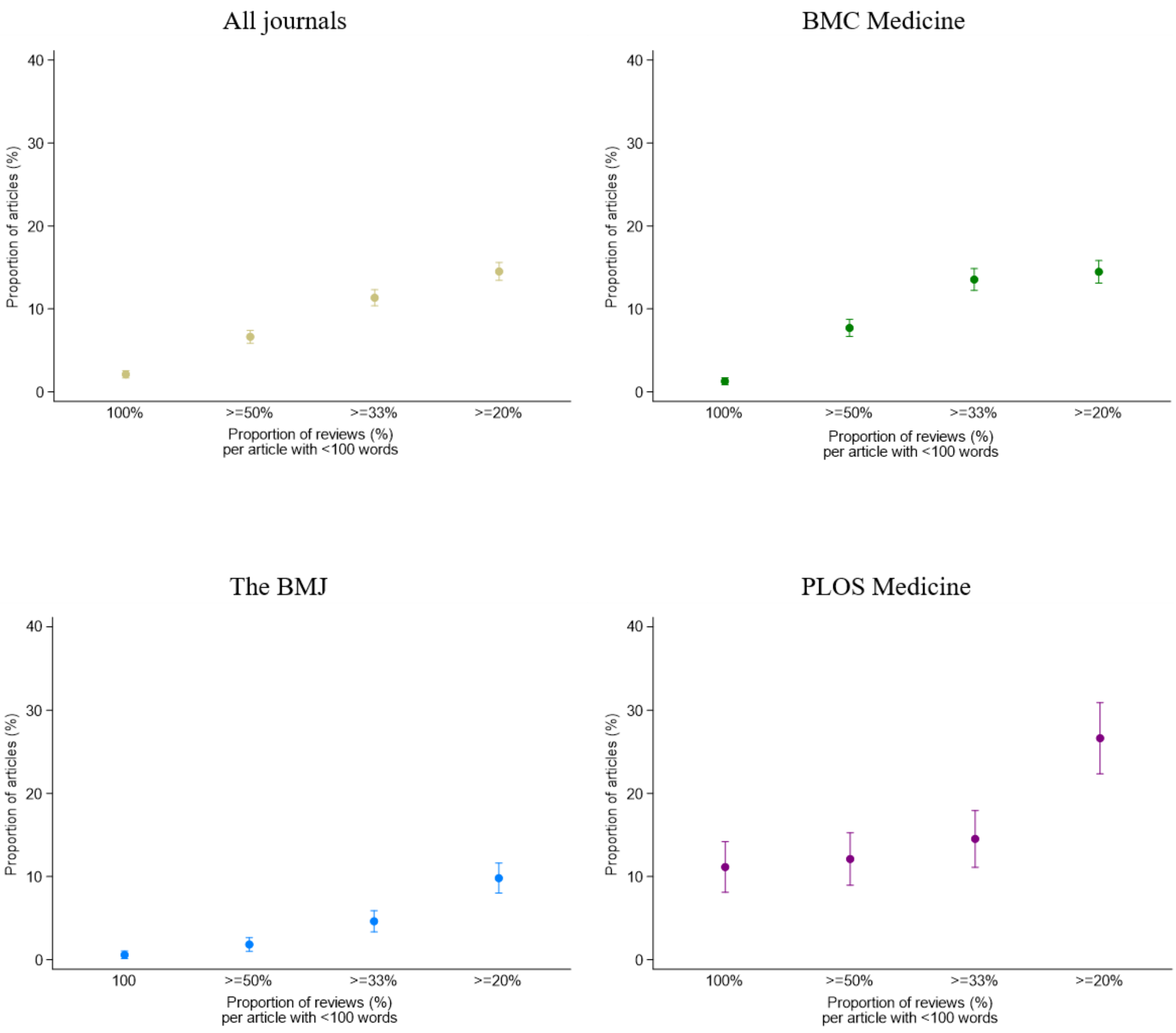

eFigure 7: Proportion of articles for which the first editorial decision was made based on a set of peer reviews consisting of 100%, ≥50%, ≥33%, or ≥20% of reviews with <300 words

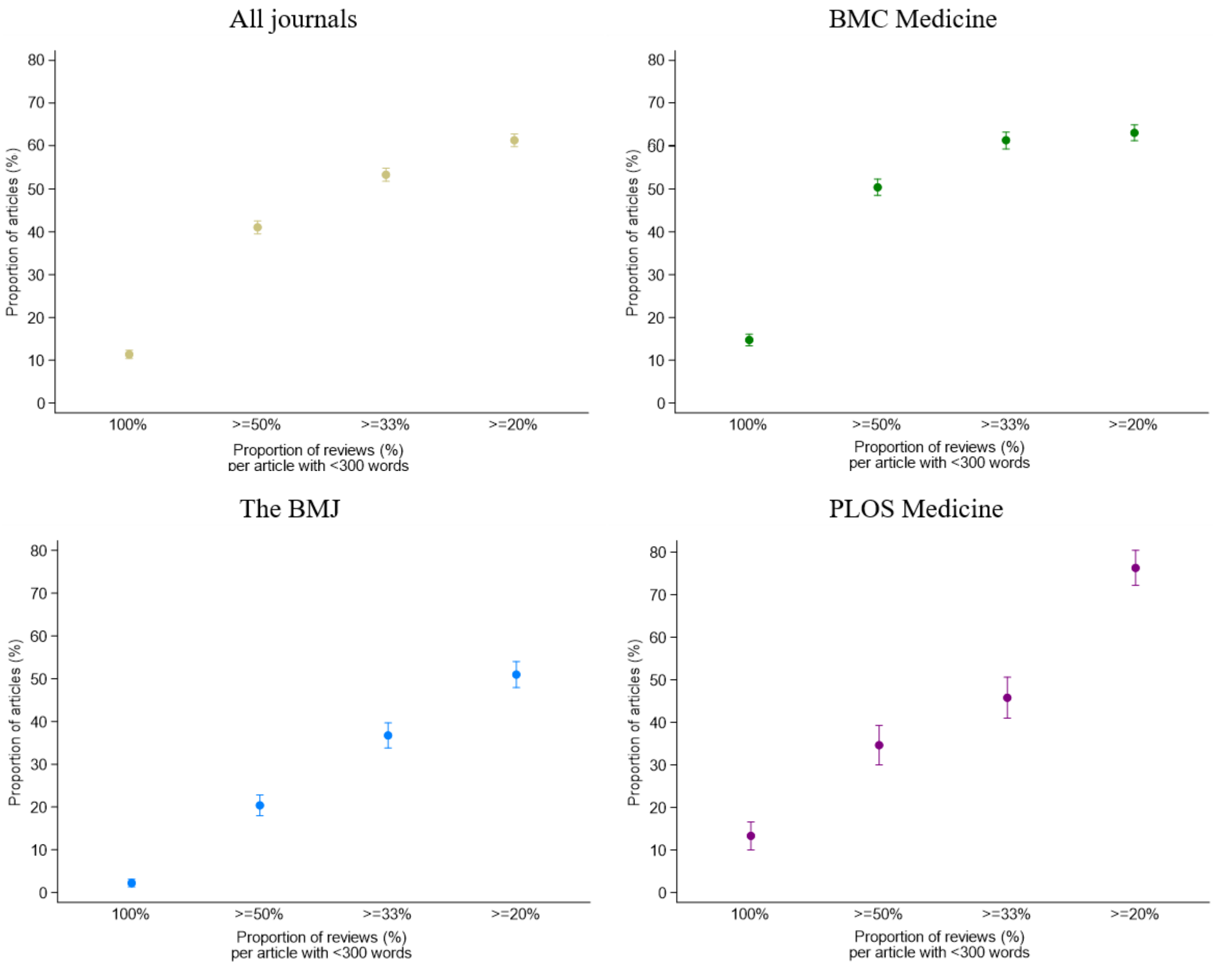

Supplement: Supplement 1. — eFigure 1. Distribution of the Number of Words Per Peer Review and the Number of Peer Reviews Per Research Article by Journal eFigure 2. Distribution of the Number of Words Per Peer Review and the Number of Peer Reviews Per Research Article for BMC Medicine Between 2003-2008 eFigure 3. Distribution of the Number of Words Per Peer Review and the Number of Peer Reviews Per Research Article for BMC Medicine Between 2009-2014 eFigure 4. Distribution of the Number of Words Per Peer Review and the Number of Peer Reviews Per Research Article For BMC Medicine and The BMJ Between 2015-2018 eFigure 5. Distribution of the Number of Words Per Peer Review and the Number of Peer Reviews Per Research Article for BMC Medicine, The BMJ, and PLOS Medicine Between 2019-2022 eFigure 6. Proportion of Articles for Which the First Editorial Decision Was Made Based on a Set of Peer Reviews Consisting of 100%, ≥50%, ≥33%, or ≥20% of Reviews With <100 Words eFigure 7. Proportion of Articles for Which the First Editorial Decision Was Made Based on a Set of Peer Reviews Consisting of 100%, ≥50%, ≥33%, or ≥20% of Reviews With <300 Words [file jamanetwopen-e2347607-s001.pdf]
